# Quadratic trigonometric B-spline for image interpolation using GA

**DOI:** 10.1371/journal.pone.0179721

**Published:** 2017-06-22

**Authors:** Malik Zawwar Hussain, Samreen Abbas, Misbah Irshad

**Affiliations:** 1 Department of Mathematics, University of the Punjab, Lahore, Pakistan; 2 Department of Mathematics, Lahore College for Women University, Lahore, Pakistan; Kaohsiung Medical University, TAIWAN

## Abstract

In this article, a new quadratic trigonometric B-spline with control parameters is constructed to address the problems related to two dimensional digital image interpolation. The newly constructed spline is then used to design an image interpolation scheme together with one of the soft computing techniques named as Genetic Algorithm (GA). The idea of GA has been formed to optimize the control parameters in the description of newly constructed spline. The Feature SIMilarity (FSIM), Structure SIMilarity (SSIM) and Multi-Scale Structure SIMilarity (MS-SSIM) indices along with traditional Peak Signal-to-Noise Ratio (PSNR) are employed as image quality metrics to analyze and compare the outcomes of approach offered in this work, with three of the present digital image interpolation schemes. The upshots show that the proposed scheme is better choice to deal with the problems associated to image interpolation.

## 1. Introduction

Trigonometric polynomials and splines have always been beneficial in multifarious research areas such as medicines, electronics [1] and computer aided geometric design [2]. Recently trigonometric polynomials have attained pronounced attention in the field of image processing in particular to investigate problems related to image compression [3]. Trigonometric splines were first introduced by Schoenberg [4] with a recurrence relation established by Lyche and Winther [5] for trigonometric B-splines of arbitrary order. Furthermore Walz [6] discussed that trigonometric B-splines of odd order form a partition of a constant in case of equidistant knots. Keeping such utilities and application potentialities of trigonometric functions in view, several new quadratic and cubic trigonometric polynomials have been introduced by researchers later on [7–9].

In early years of developments in the field of image processing a variety of spline functions with confusing naming were found. Sometimes they are referred as cubic splines [10], bicubic splines [11] or high resolution splines [12], while interpolation is named as cubic convolution interpolation [13]. Hou and Andrews [10] for the first time discussed the computational implementation of B-splines as a tool in various digital image and signal processing applications both software and hardware point of view. The work was then extended by Unser in [14]. Parker, Kenyon and Troxel [12] studied comparison of different interpolation function with B-spline interpolation to preserve the quality of digital images. Luu, Wang, Vo, et al. [15] presented an improved interpolation method to enhance quality of two dimensional digital images with family of recursive interpolation functions based on B-spline representation and its inverse gradient weighting version.

In recent year some state-of-the-art image interpolation techniques became popular among the community of image processing. Li and Orchard [16] developed an edge-directed interpolation algorithm to estimate local covariance coefficients from a low-resolution image and adapt the interpolation at a higher resolution based on the geometric duality between their covariance. Li in [17] established a patch-based image interpolation algorithm under a set theoretic framework which formed as an alternate to the projection onto two convex sets: set of observation data and the other defined by a sparsity-based nonlocal prior. Hung and Siu [18] proposed a single frame super resolution algorithm using a finite impulse response; Wiener-filter. The correlation matrices are estimated using the nonlocal means filter to form a new iterative framework which is used to improve the estimated correlation and estimated high-resolution image.

Although B-spline were constructed as convolution of certain probability distributions in early nineteenth century, Schoenberg [19] in 1946 used them for statistical data smoothness which started a new era of modern mathematical study of spline approximation. Later on Lewis [20] for the first time developed local support basis for *GC*^2^ splines. Bartels and Beatty [21] constructed local basis functions for *β*-spline. Foley [22] developed a B-spline like basis for different weights spline such as *ν*-spline, *β*-spline and *γ*-spline. Sarfraz [23] established number of B-spline like local basis for the rational splines. These splines maintains the geometrical smoothness, continuity and computational simplicity achieved by other *ν*-spline, *β*-spline, *γ*-spline and weighted splines.

In this paper, an iterative image interpolation scheme is designed to investigate the two dimensional digital image interpolation problems. First a quadratic trigonometric B-spline with control parameters is established using a constructive approach described in [24], then an optimization technique based on Genetic Algorithm (GA) helps find the appropriate values of control parameters in the description of B-spline. The interpolation scheme is made up of several stages to achieve the resulting interpolated images. Gray scale digital images are considered as reference image into account. Moreover to analyze the quality of resulting interpolated images FSIM, SSIM, MS-SSIM indices and PSNR are utilized as standard image quality metrics. A comparison of the outcomes of proposed interpolation scheme with three of the state-of-the-art image interpolation schemes is also made.

Rest of the paper is organized as follows. Section 2 is about the construction of local support quadratic trigonometric B-spline basis with control parameters defined in its description. Section 3 is dedicated to overall methodology and proposed image interpolation scheme. Demonstration of the proposed interpolation scheme is presented in Section 4. Finally, Section 5 concludes the paper.

## 2. Quadratic trigonometric B-spline

A *C*^1^ quadratic trigonometric spline is proposed here to establish new local support quadratic B-spline basis which consists of two control parameters in its depiction. Quadratic B-spline basis are attained by a transformation to piecewise defined Bernstein-Bézier form which will then be used to formulate quadratic trigonometric B-spline representation.

### 2.1 Quadratic trigonometric spline

**Fig 1:**
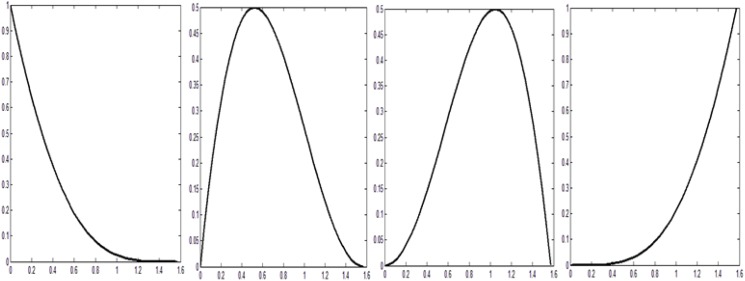
Quadratic trigonometric basis function.

Let (*x*_*i*_,*F*_*i*_), *i* = 1,2,…,*n* be the given set of data demarcated over the interval [a*,b*] where the partition of the data is a* = *x*_0_ < *x*_1_ < *x*_2_ < ⋯ < *x*_*n*_ = b*. A piecewise quadratic trigonometric spline function *T*(*x*) with parameters *α*_*i*_,*β*_*i*_ ∈ [−1,1] over each sub interval I*_i_ = [*x*_*i*_,*x*_*i*+1_]; *i* = 1,2…,*n*, is defined as:

$${T|}_{\left[x_{i},x_{i+1}\right]}(x)=T_{i}(x)=\sum_{l=0}^{3}S_{l,i}(\varphi ,\alpha_{i},\beta_{i})Q_{l}\;,\;\forall x\in [x_{i},x_{i+1}]$$ (1)

where *Q*_*l*_; *l* = 0,1,2,3, are co-efficient of interpolation which are defined as follows:

$$Q_{0}=F_{i},\;Q_{1}=F_{i}+h_{i}D_{i}/(1+\alpha_{i})\frac{\pi }{2},$$

$$Q_{2}=F_{i+1}-h_{i}D_{i+1}/(1+\beta_{i})\frac{\pi }{2},\;Q_{3}=F_{i+1},$$

with *h*_*i*_ = *x*_*i*+1_ − *x*_*i*_. *D*_*i*_ are the derivatives at the knots *x*_*i*_ which may be given or estimated using any approximation schemes; geometric mean, arithmetic mean and harmonic mean. Furthermore, the quadratic trigonometric spline function (1) has the following *C*^1^ interpolating properties:

$$T\left(x_{i}\right)=F_{i}\;\mathrm{and}\;T^{\left(1\right)}\left(x_{i}\right)=D_{i},\;i=0,1,\ldots ,n$$ (2)

where *T*^(1)^(*x*) represents the derivatives with respect to the variable *x*. The functions *S*_*l*,*i*_(*φ*,*α*_*i*_,*β*_*i*_); *l* = 0,1,2,3 in (1) are the quadratic trigonometric basis functions defined in each sub intervals [*x*_*i*_,*x*_*i*+1_] as:

$$\begin{array}{c} S_{0,i}(\varphi ,\alpha_{i},\beta_{i})=(1-sin\varphi )(1-\alpha_{i}sin\varphi )\\ S_{1,i}(\varphi ,\alpha_{i},\beta_{i})=(1+\alpha_{i})sin\varphi (1-sin\varphi )\\ S_{2,i}(\varphi ,\alpha_{i},\beta_{i})=(1+\beta_{i})cos\varphi (1-cos\varphi )\\ S_{3,i}(\varphi ,\alpha_{i},\beta_{i})=(1-cos\varphi )(1-\beta_{i}cos\varphi ) \end{array}\}$$ (3)

with $\varphi =\frac{\pi }{2}\delta$ and $\delta =(\frac{x-x_{i}}{h_{i}})$. From Eq (3) it can be observed that the quadratic trigonometric functions are Bernstein Bézier like basis functions which are demonstrated in Fig 1.

Moreover these quadratic functions satisfied the following properties:

1. Nonnegative: *S*_*l*,*i*_(*φ*,*α*_*i*_,*β*_*i*_) ≥ 0; *l* = 0,1,2,3, *i* = 1,2,…,*n*−1.
2. Partition of unity: $\sum_{l=0}^{3}S_{l,i}(\varphi ,\alpha_{i},\beta_{i})=1\;;\;i=1,2,\ldots ,n-1$.
3. Symmetry: *S*_*l*,*i*_(*φ*,*α*_*i*_,*β*_*i*_) = *S*_3−*l*,*i*_(*φ*,*β*_*i*_,*α*_*i*_); *l* = 0,1,2,3, *i* = 1,2,…,*n*−1.
4. Convex hull property: The entire segments of piecewise quadratic trigonometric spline function must lie inside the control polygon spanned by *P*_0_,*P*_1_,*P*_2_ and *P*_3_.
5. Variation Diminishing Property: It can be stated as the curve has no more intersections with any plane than does the control polygon.

**Fig 2:**
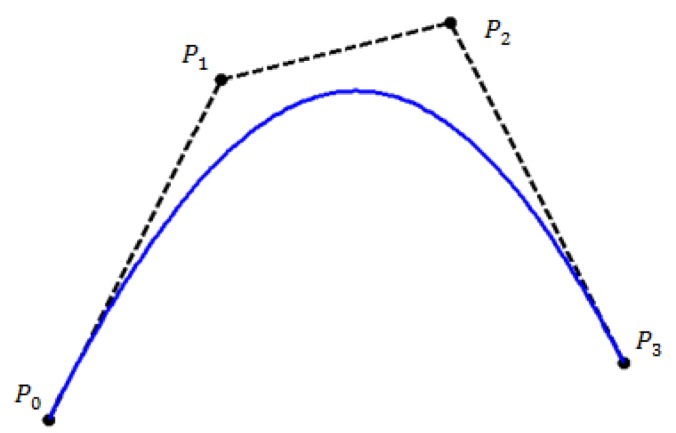
Graphical view of convex hull property for quadratic trigonometric spline function.

**Fig 3:**
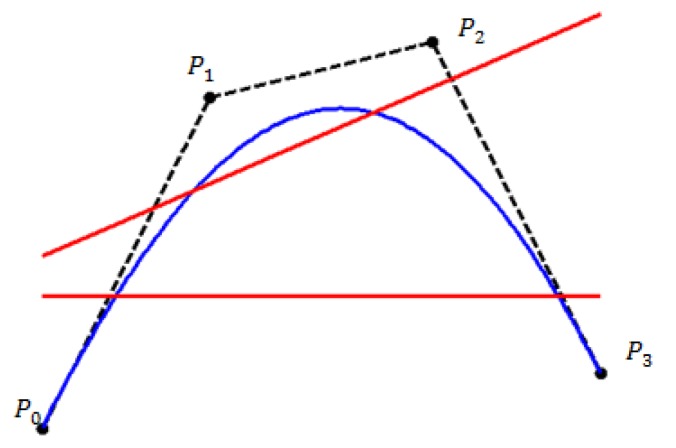
Graphical view of variation diminishing property for quadratic trigonometric spline function.

Figs 2 and 3 respectively show the graphical view of convex hull property and the variation diminishing property for the proposed quadratic trigonometric spline function.

### 2.2 Local support quadratic trigonometric B-spline basis

On the way to construct local support quadratic trigonometric B-spline basis, additional knots *x*_−3_ < *x*_−2_ < *x*_−1_ < *x*_0_ and *x*_*n*_ < *x*_*n*+1_ < *x*_*n*+2_ < *x*_*n*+3_ will be introduced on both outsides of the interval [*x*_0_,*x*_*n*_] with control parameters *α*_*i*_, *β*_*i*_ > 0; *i* = −3, −2, …, *n* + 3, defined on this stretched partition of [*x*_0_,*x*_*n*_]. A quadratic spline *ϑ*_*l*_(*x*); *l* = −1, …, *n* + 3, can be defined as:

$$\vartheta_{l}(x)=\left\{ \begin{array}{c} 0\;\mathrm{for}\;x<x_{l-2},\\ 1\;\mathrm{for}\;x\geq x_{l+1.} \end{array}\right.$$ (4)

Therefore, the function *ϑ*_*l*_(*x*) will have the following quadratic form for the remaining intervals [*x*_*i*_,*x*_*i*+1_), *i* = *l* − 2,*l* − 1,*l*.

$$\vartheta_{l}(x)=S_{0,i}(\varphi ,\alpha_{i},\beta_{i})\vartheta_{l}(x_{i})+S_{1,i}(\varphi ,\alpha_{i},\beta_{i})\left(\vartheta_{l}(x_{i})+\frac{h_{i}}{(1+\alpha_{i})\frac{\pi }{2}}{\vartheta_{l}}^{\left(1\right)}(x_{i})\right)+S_{2,i}(\varphi ,\alpha_{i},\beta_{i})\left(\vartheta_{l}(x_{i+1})-\frac{h_{i}}{(1+\beta_{i})\frac{\pi }{2}}{\vartheta_{l}}^{\left(1\right)}(x_{i+1})\right)+S_{3,i}(\varphi ,\alpha_{i},\beta_{i})\vartheta_{l}(x_{i+1})$$ (5)

where *S*_*l*,*i*_(*φ*,*α*_*i*_,*β*_*i*_); *l* = 0,1,2,3 are same as defined in Eq (3). The requirement that *ϑ*_*l*_ is continuous up to second order in particular at *x*_*l*−2_, *x*_*l*−1_, *x*_*l*_ and *x*_*l*+1_, the quadratic trigonometric function uniquely determined the following

$$\begin{array}{l} \vartheta_{l}\left(x_{l-2}\right)=0,\;\vartheta_{l}{}^{\left(1\right)}\left(x_{l-2}\right)=0,\\ \;\vartheta_{l}\left(x_{l-1}\right)=\mu_{l-1},\;\vartheta_{l}{}^{\left(1\right)}\left(x_{l-1}\right)={\hat{\mu }}_{l-1},\\ \;\vartheta_{l}\left(x_{l}\right)=1-\lambda_{l},\;\vartheta_{l}{}^{\left(1\right)}\left(x_{l}\right)={\hat{\lambda }}_{l},\\ \vartheta_{l}\left(x_{l+1}\right)=1,\;\vartheta_{l}{}^{\left(1\right)}\left(x_{l+1}\right)=0, \end{array}\}$$ (6)

where

$$\begin{array}{l} \mu_{l}=h_{l-1}\;{\hat{\mu }}_{l}/\pi ,\;{\hat{\mu }}_{l}=h_{l-1}d_{l+1}/c_{l+1},\\ \lambda_{l}=h_{l}{\hat{\lambda }}_{l}/\pi ,\;{\hat{\lambda }}_{l}=h_{l}d_{l-1}/c_{l},\\ d_{l}=\frac{{2h}_{l-1}}{(1+\alpha_{l-1})\pi }+\frac{2h_{l}}{(1+\beta_{l})\pi }, \end{array}$$

and

$$c_{l}=h_{l}d_{l}(\frac{{2h}_{l-1}}{(1+\alpha_{l-1})\pi }+\frac{2h_{l-2}}{(1+\beta_{l-2})\pi })+h_{l-1}d_{l-1}(\frac{{2h}_{l-1}}{(1+\alpha_{l-1})\pi }+\frac{2h_{l}}{(1+\beta_{l})\pi })+h_{l-2}d_{l}d_{l-1}.$$

**Fig 4:**
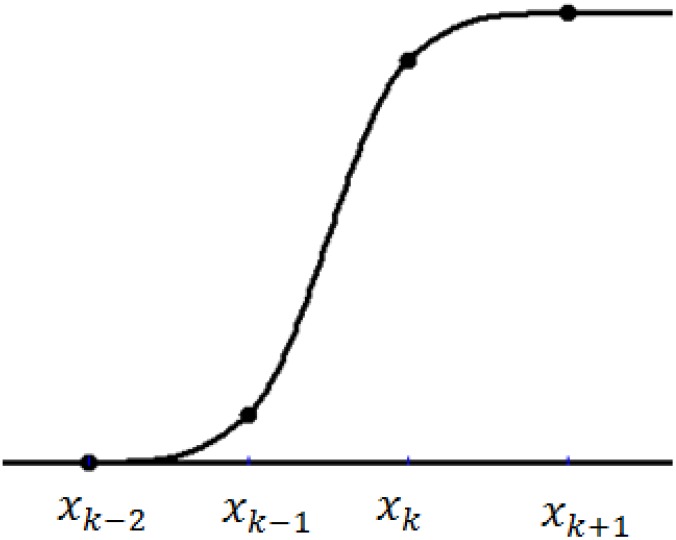
The quadratic trigonometric spline *ϑ*_*l*_(*x*).

The graphical view of the quadratic trigonometric function *ϑ*_*l*_(*x*), *l* = −1,0,…,*n* + 2, is shown in Fig 4.

Next the local support quadratic trigonometric B-spline basis is elaborated by the difference of the quadratic functions *ϑ*_*l*_ as:

$$B_{l}\left(x\right)=\vartheta_{l}\left(x\right)-\vartheta_{l+1}\left(x\right),\;l=-1,\ldots ,n+1$$ (7)

Therefore, an explicit form of the quadratic trigonometric B-spline *B*_*l*_(*x*) on any interval [*x*_*i*_,*x*_*i*+1_) is computed from (4)–(7) as:

$$B_{l}(x)=S_{0,i}(\varphi {,\alpha }_{i},\beta_{i})B_{l}(x_{i})+S_{1,i}(\varphi ,\alpha_{i},\beta_{i})\left(B_{l}(x_{i})+\frac{h_{i}}{(1+\alpha_{i})\frac{\pi }{2}}{B_{l}}^{\left(1\right)}(x_{i})\right)+S_{2,i}(\varphi ,\alpha_{i},\beta_{i})\left(B_{l}(x_{i+1})-\frac{h_{i}}{(1+\beta_{i})\frac{\pi }{2}}{B_{l}}^{\left(1\right)}(x_{i+1})\right)+S_{3,i}(\varphi ,\alpha_{i},\beta_{i})B_{l}(x_{i+1})$$ (8)

where *B*_*l*_(*x*_*i*_) = *B*_*l*_^(1)^(*x*_*i*_) = 0, for *i* ≠ *l* − 1, *l*, *l* + 1

$$\begin{array}{l} B_{l}(x_{l-1})=\mu_{l-1}\;,\;{B_{l}}^{\left(1\right)}(x_{l-1})={\hat{\mu }}_{l-1},\\ B_{l}(x_{l})={1-\lambda_{l}-\mu }_{l}\;,\;{B_{l}}^{\left(1\right)}(x_{l})={\hat{\lambda }}_{l}-{\hat{\mu }}_{l},\\ B_{l}(x_{l+1})=\lambda_{l+1},\;\mathrm{and}\;{B_{l}}^{\left(1\right)}(x_{l+1})=-{\hat{\lambda }}_{l+1}. \end{array}$$

Hence, it can be noticed that the quadratic trigonometric B-spline basis *B*_*l*_(*x*), *l* = −1, −2, …, *n* + 1, have the following properties:

1. *B*_*l*_(*x*) = 0,              for *x* ∉ (*x*_*l*−2_,*x*_*l*+2_)
2. $\sum_{l=-1}^{n+1}B_{l}(x)=1$,      for *x* ∈ [*x*_0_,*x*_*n*_]
3. *B*_*l*_(*x*_*l*_) > 0,              for all *x*.

**Fig 5:**
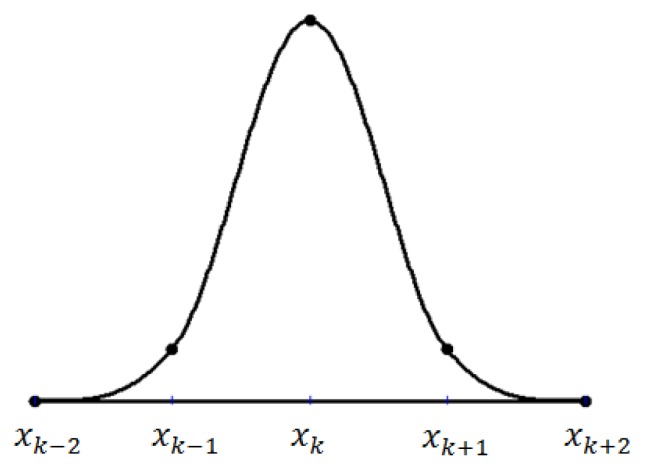
The quadratic trigonometric B-spline B_l_(x).

The graphical view of the quadratic trigonometric B-spline basis function *B*_*l*_(*x*), *l* = −1,0,…,*n* + 1, is shown in Fig 5.

### 2.3 1D and 2D quadratic trigonometric B-spline functions

In this section, 1D and 2D quadratic trigonometric B-spline functions are established. Both the functions are inspired by the local support basis (8). Let T*(*x*) be the desired 1D quadratic trigonometric B-spline interpolating function,

$$T^{*}(x)=\sum_{l=-1}^{n+1}B_{l}(x)Q_{l}^{*}\;,\;\forall x\in [x_{0},x_{n}],$$ (9)

where $Q_{l}^{*}$ are the co-efficient of interpolation and can be obtained from the set of discrete data defined at certain given spatial points. Furthermore from the local support properties defined in Section 2.2, Eq (9) can be rewrite as

$$T^{*}(x)=\sum_{l=i-1}^{i+2}B_{l}(x)Q_{l}^{*}\;,\;\forall x\in [x_{i},x_{i+1}),\;i=0,1,\ldots ,n-1.$$ (10)

Thus by substituting the values of *B*_*l*_(*x*) from Eq (8) in Eq (10)

$$T^{*}(x)=S_{0,i}(\varphi ,\alpha_{i},\beta_{i})F_{l}+S_{1,i}(\varphi ,\alpha_{i},\beta_{i})V_{l}+S_{2,i}(\varphi ,\alpha_{i},\beta_{i})W_{l}+S_{3,i}(\varphi ,\alpha_{i},\beta_{i})F_{l+1}$$ (11)

where $F_{l}=\lambda_{l}Q_{l-1}^{*}+{(1-\lambda_{l}-\mu }_{l})Q_{l}^{*}+\mu_{l}Q_{l+1}^{*}$,

$$\begin{array}{l} V_{l}={(1-\sigma }_{l})\lambda_{l}Q_{l-1}^{*}+\{1-{(1-\sigma }_{l})\lambda_{l}-{(1+\sigma }_{l})\mu_{l}\}Q_{l}^{*}+{(1+\sigma }_{l})\mu_{l}Q_{l+1}^{*},\\ W_{l}={(1+\omega }_{l})\lambda_{l}Q_{l}^{*}+\{1-{(1+\omega }_{l})\lambda_{l}-{(1-\omega }_{l})\mu_{l}\}Q_{l+1}^{*}+{(1-\omega }_{l})\mu_{l}Q_{l+2}^{*} \end{array}$$

with *σ*_*l*_ = 2/(1 + *σ*_*l*_), and *ω*_*l*_ = 2/(1 + *β*_*l*_).

As Eq (11) represents the piecewise Bernstein-Bézier form of quadratic trigonometric B-spline so the function T*(*x*) can be rewritten in vector form as:

$$T^{*}\left(x\right)=\sum_{l=0}^{3}S_{l,i}\left(\varphi ,\alpha_{i},\beta_{i}\right)X_{l}\;,\;\forall \;x\in [x_{i},x_{i+1}];\;i=0,1,\ldots ,n-1$$ (12)

where *X*_*l*_ = [*F*_*l*_ *V*_*l*_ *W*_*l*_ *F*_*l*+1_]^*T*^ and *X*_*l*_ = *Y*_*l*_*Z*_*l*_ with

$$Y_{l}=\left[\begin{array}{cccc} \lambda_{l} & \left(1-\lambda_{l}-\mu_{l}\right) & \mu_{l} & 0\\ (1-\sigma_{l})\lambda_{l} & \left\{1-(1-\sigma_{l})\lambda_{l}-(1+\sigma_{l})\mu_{l}\right\} & (1+\sigma_{l})\mu_{l} & 0\\ 0 & (1+\omega_{l})\lambda_{l} & \left\{1-(1+\omega_{l})\lambda_{l}-(1-\omega_{l})\mu_{l}\right\} & (1-\omega_{l})\mu_{l}\\ 0 & \lambda_{l+1} & 1-\lambda_{l+1}-\mu_{l+1} & \mu_{l+1} \end{array}\right]$$

$$Z_{k}={\left[\begin{array}{cccc} Q_{l-1}^{*} & Q_{l}^{*} & Q_{l+1}^{*} & Q_{l+2}^{*} \end{array}\right]}^{T}.$$

In order to extend the above 1D B-spline interpolating function to two dimensions, Eq (9) can be written as:

$$T^{*}\left(x,\tilde{x}\right)=\sum_{l=-1}^{n+1}\sum_{k=-1}^{m+1}B_{l}\left(x\right){\tilde{B}}_{k}\left(\tilde{x}\right)Q_{l,k}^{*}\;,\;\forall x\in [x_{0},x_{n}],\;\tilde{x}\in [{\tilde{x}}_{0},{\tilde{x}}_{m}],$$ (13)

where *B*_*l*_(*x*) are same as defined in Section 2.2 and analogously ${\tilde{B}}_{k}(\tilde{x})$ are the quadratic trigonometric B-spline basis corresponding to a set of knots ${\tilde{x}}_{j},j=-3,-2,\ldots ,m+3$. Here the control parameters ${\tilde{\alpha }}_{j},{\tilde{\beta }}_{j};\;j=-2,-1,\ldots m+2$ also correspond to the set of knots ${\tilde{x}}_{j},j=-3,-2,\ldots ,m+3$. Hence for *x* ∈ [*x*_*i*_,*x*_*i*+1_], $\tilde{x}\in [{\tilde{x}}_{j},{\tilde{x}}_{j+1}]$, the function $T^{*}(x,\tilde{x})$ be the required 2D quadratic trigonometric B-spline in vector form is defined as:

$$T^{*}(x,\tilde{x})=\sum_{l=0}^{3}\sum_{k=0}^{3}S_{l,i}(\varphi ,\alpha_{i},\beta_{i})\;S_{k,j}(\tilde{\varphi },{\tilde{\alpha }}_{j},{\tilde{\beta }}_{j})\;X_{l,k}^{i,j}$$ (14)

where the points $X_{l,k}^{i,j}$ can be computed from the quadratic trigonometric B-spline vertices $Q_{l,k}^{*}$ as

$$X_{l,k}^{i,j}=Y_{l}Z_{i,j}{\tilde{Y}}_{k}^{T}$$ (15)

where

$$X_{l,k}^{i,j}=\left[\begin{array}{cccc} X_{0,0}^{i,j} & X_{0,1}^{i,j} & \cdots & X_{0,k}^{i,j}\\ X_{1,0}^{i,j} & \cdots & \cdots & \cdots \\ \cdots & \cdots & \cdots & \cdots \\ X_{l,0}^{i,j} & \cdots & \cdots & X_{l,k}^{i,j} \end{array}\right],$$

$$Z_{i,j}=\left[\begin{array}{cccc} Q_{i-1,j-1}^{*} & Q_{i-1,j}^{*} & Q_{i-1,j+1}^{*} & Q_{i-1,j+2}^{*}\\ Q_{i,j-1}^{*} & Q_{i,j}^{*} & Q_{i,j+1}^{*} & Q_{i,j+2}^{*}\\ Q_{i+1,j-1}^{*} & Q_{i+1,j}^{*} & Q_{i+1,j+1}^{*} & Q_{i+1,j+2}^{*}\\ P_{i+2,j-1}^{*} & Q_{i+2,j}^{*} & Q_{i+2,j+1}^{*} & Q_{i+2,j+2}^{*} \end{array}\right],$$

and the matrix *Y*_*l*_ is given in (12) with a corresponding extension for ${\tilde{Y}}_{k}$.

## 3. Genetic algorithm approach to image interpolation

This section describes the overall process of implementation of soft computing technique genetic algorithm to image interpolation using newly constructed quadratic trigonometric B-spline. The process includes the phases of problem matching with genetic algorithm and image interpolation based on quadratic trigonometric B-spline basis. An introduction to the parameters used for genetic algorithm is also made a part to this section.

### 3.1 Optimization by genetic algorithm

Genetic algorithms [25] are probabilistically modeled search techniques which are used to find out global optimal solutions in an effectual way as compare to other conventional optimization techniques even in large solution spaces. Genetic algorithms represent a class of parallel adaptive search techniques whose mechanisms are based on natural selection of optimal solutions through some biological evolutionary procedure. They use multiple search points at the same time to find out an optimal solution without going through an exhaustive mechanism of searching.

**Fig 6:**
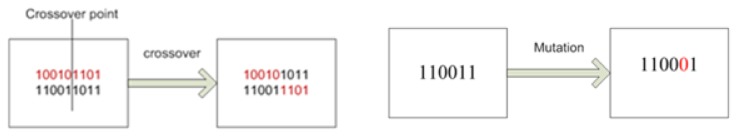
Crossover and the mutation operators for genetic algorithm.

Genetic algorithms start with a collection of solutions which are analogous to the chromosomes in natural systems of genetics. Each chromosome usually represents a binary bit string with individual bits 0 or 1which are named as genes. The collection of candidate solution is called population which is randomly chosen from the solution space at the beginning when the search through genetic algorithms starts. The main three genetic operators ‘selection’, ‘crossover’ and ‘mutation’ help genetic algorithms for survival in the large solution space. The crossover and the mutation operators are exemplified here in Fig 6 to illustrate the overall process of genetic algorithm for some selected chromosomes.

As this work is aimed to search an optimal solution for image interpolation based on quadratic trigonometric B-spline basis with control parameters $\alpha_{i},{\tilde{\alpha }}_{j}{,\beta }_{i}$, and ${\tilde{\beta }}_{j}$ for *i* = 0,1,2,…,*n* − 1 and *j* = 0,1,2,…,*m* − 1. Therefore, genetic algorithm is employed here to pursue the appropriate values of control parameters. Now before starting the discussion on genetic algorithm, several terms and system parameters are needed to be defined in advance. In this work the maximum no. of iterations or generations set is 10 and the size of population taken is 20. Initially population is generated by randomly selecting chromosomes or bit strings where each single gene represents the value for control parameters $\alpha_{i},{\tilde{\alpha }}_{j}{,\beta }_{i}$, and ${\tilde{\beta }}_{j}$ as defined in the portrayal of quadratic trigonometric B-spline (14). Moreover a fitness or objective function is the sum square error which is defined for the image spatial data and formulated as:

$$E_{ij}(\alpha_{i},{\tilde{\alpha }}_{j}{,\beta }_{i},{\tilde{\beta }}_{j})=\sum_{i}\sum_{j}{\left[{Im}_{ij}^{\prime }(\alpha_{i},{\tilde{\alpha }}_{j}{,\beta }_{i},{\tilde{\beta }}_{j})-{Im}_{ij}\right]}^{2}$$ (16)

where *Im*_*ij*_ and ${Im}_{ij}^{\prime }$ represent the original reference image and resulting interpolated images respectively. Genetic operations selection, crossover and mutation will be used to maintain the preeminence of optimal solution. The process of genetic algorithm will be stopped when the predefined number of iterations is attained.

### 3.2 Proposed image interpolation scheme

**Fig 7:**
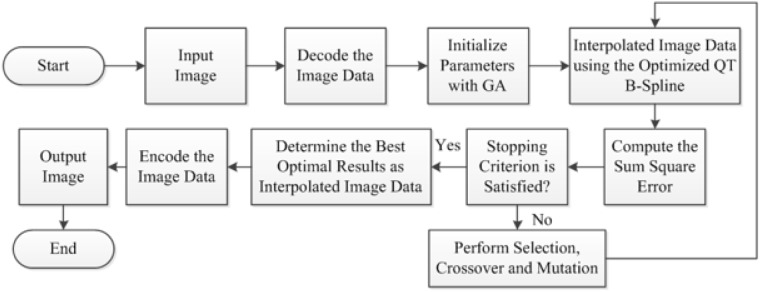
Flow diagram of proposed image interpolation scheme.

In this section, an image interpolation scheme is designed using quadratic trigonometric B-spline (15) and genetic algorithm. The scheme comprises of several steps which are elaborated here one by one. First of all, it finds the spatial data of a selected reference image through some image decoding schemes. Secondly, all the system parameters of GA are initialized to attain the optimal values of control parameters $\alpha_{i},{\tilde{\alpha }}_{j}{,\beta }_{i}$, and ${\tilde{\beta }}_{j}$. An initial population is made randomly with possible combination of the values of parameters. Successive implementations of search operations mutation, crossover and selection to this population make a better lead to optimal values of $\alpha_{i},{\tilde{\alpha }}_{j}{,\beta }_{i}$, and ${\tilde{\beta }}_{j}$ such that the sum square error (16) is minimized. Thus the optimal values of $\alpha_{i},{\tilde{\alpha }}_{j}{,\beta }_{i}$, and ${\tilde{\beta }}_{j}$ make the whole quadratic trigonometric B-spline representation optimized and the interpolated image designed using the optimized B-spline will be called optimal interpolated image. So, finally the image data is interpolated using the optimized quadratic trigonometric B-spline. Repeated implementations of search operations for respective iterations of GA will help to contain better approximation until the best optimal resulting interpolated image is obtained. Fig 7 presents the flow diagram of the proposed scheme.

## 4. Experimental results

In this section, the proposed quadratic trigonometric image interpolation scheme with soft computing technique, genetic algorithm, defined in Section 3 is tested and compared with some representative work in the literature: the image Super Resolution algorithm based on Non-Local Means (SR-NLM) [18], Patch Based Non-Local (PB-NL) image interpolation [17] and New Edge-Directed Interpolation (NEDI) [16]. The proposed scheme is tested on three different natural images. The images are gray scaled in 512×512 resolution with eight bits per pixel and named as ‘Moon-Surface’, ‘Boat’ and ‘Truck’ respectively. The images were down-sampled first to acquire low-resolution images and then up-sampled twice using the proposed image interpolation scheme to get the resulting interpolated images. For SR-NLM, PB-NL and NEDI implementation codes are provided by the authors and available online at [26, 27].

As the original test images are known so one can easily characterized the difference between the resulting and original reference images using different existing image quality metrics. Structure SIMilarity (SSIM) index [28], Multi-Scale Structure SIMilarity (MS-SSIM) index [29] and Feature SIMilarity (FSIM) index [30] along with the traditional Peak Signal-to-Noise Ratio (PSNR) [31] are utilized here to assess the expediency of proposed optimal image interpolation scheme. PSNR is a classical objective image quality metric to measure the intensity differences of two images. It is usually measured in the unit of decibels (dB) and attains high values if an image has a high picture quality and attains a low value if an image contains a low picture quality but in some examples the resulting images may look close to the original reference images though they have low PSNR values. Whereas SSIM, MS-SSIM and FSIM indices take values in [0,1] where 1 is only reachable in the case when both the original and the resulting images are identical.

**Fig 8:**
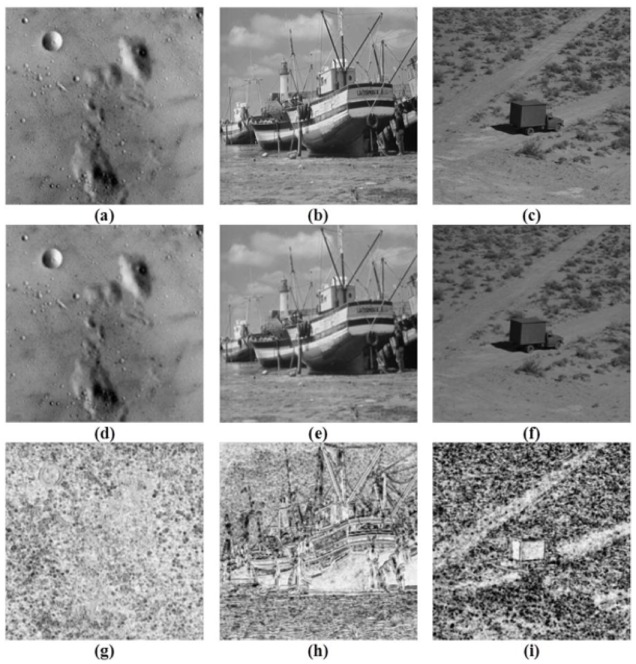
Original test images (a) ‘Moon-Surface’ (b) ‘Boat’ (c) ‘Truck’, Resulting images interpolated by using proposed Quadratic Trigonometric B-Spline representation and GA (d) ‘Moon-Surface’ (e) ‘Boat’ (f) ‘Truck’, SSIM map of interpolated images (g) ‘Moon-Surface’ (h) ‘Boat’ (i) ‘Truck’.

Tables 1–4 depict the PSNR, SSIM, MS-SSIM and FSIM values respectively for the proposed image interpolation scheme and the existing schemes like: SR-NLM, NEDI and PB-NL. The outcomes indicate that the proposed image interpolation scheme yields better results than the other three schemes for the test images. Moreover Fig 8 shows the subjective view of the resulting image with their SSIM maps where brightness indicates the magnitude of the SSIM index near to 1 that is if the SSIM map of an image is brighter the image has a better quality.

**Table 1:** Comparison of different interpolation schemes using PSNR values.

Interpolation Schemes	Moon-Surface	Boat	Truck
NEDI	33.9304	26.9721	31.2363
PB-NL	33.7652	26.0944	30.1622
SR-NLM	33.0563	27.0247	30.6924
Proposed Scheme	**36.0932**	**27.6615**	**31.7427**

**Table 2:** Comparison of different interpolation schemes using SSIM values.

Interpolation Schemes	Moon-Surface	Boat	Truck
NEDI	0.8990	0.7583	0.8275
PB-NL	0.8970	0.7408	0.7897
SR-NLM	0.8919	0.7745	0.8039
Proposed Scheme	**0.9273**	**0.8370**	**0.8556**

**Table 3:** Comparison of different interpolation schemes using MS-SSIM values.

Interpolation Schemes	Moon-Surface	Boat	Truck
NEDI	0.9702	0.9233	0.9560
PB-NL	0.9738	0.9244	0.9462
SR-NLM	0.9705	0.9219	0.9500
Proposed Scheme	**0.9831**	**0.9642**	**0.9676**

**Table 4:** Comparison of different interpolation schemes using FSIM values.

Interpolation Schemes	Moon-Surface	Boat	Truck
NEDI	0.9776	0.9380	0.9478
PB-NL	0.9715	0.9308	0.9359
SR-NLM	0.9752	0.9358	0.9407
Proposed Scheme	**0.9799**	**0.9602**	**0.9702**

## 5. Conclusions

A newly constructed quadratic trigonometric B-spline representation and a soft computing technique genetic algorithm are combined together to design an image interpolation scheme. The image quality metrics SSIM, MS-SSIM and FSIM indices along with traditional PSNR are employed to assure the quality of resulting interpolated images. Experimental outcomes and comparison with existing image interpolation schemes show that the suggested quadratic trigonometric B-spline together with genetic algorithm is a better representation and plays a significant role for the problem related to image interpolation e.g. image magnification and geometric transformation (resizing, rotation, sharing, etc.).
